# Axillary management in the post-neoadjuvant setting: dual tracer vs. single tracer for sentinel lymph node biopsy

**DOI:** 10.1007/s13304-025-02441-5

**Published:** 2025-10-29

**Authors:** Alessia Luciana Margherita, Fabrizia Calenda, Nicola Rocco, Martina Pontillo, Carlo Luigi Junior Castiello, Mafalda Ingenito, Nunzio Velotti, Mario Musella

**Affiliations:** 1 Department of Advanced Biomedical Sciences, University of Naples “Federico II”, Via Sergio Pansini 5, 80131 Naples, Italy; 2 Breast Unit, University Hospital Federico II, Naples, Italy

**Keywords:** Breast cancer, Breast suregry, Axillary surgery, Surgical de-escalation, Sentinel lymph node biopsy

## Abstract

Significant disparities exist in axillary management between and within countries, particularly regarding patients undergoing neo-adjuvant chemotherapy (NAC) who present with nodal disease and subsequently become ycN0 after systemic treatment. The aim of this study was to evaluate the efficacy and safety of the single-tracer technique for clinically node-positive (cN +) breast cancer patients who achieve node-negativity (ycN0) after NAC and compare this approach with the dual-tracer technique. We included consecutive patients diagnosed with cT1-T2, cN1-N2 breast cancer who underwent NAC and achieved nodal disease regression (ycN0). The patients were divided into two groups: “dual tracer” technique for sentinel lymph node identification and “single tracer” technique. In both groups, axillary lymph node dissection (ALND) was performed in patients with positive sentinel lymph nodes. Out of the 76 patients included, 42 underwent sentinel lymph node removal using the dual-tracer technique, while 34 underwent the single-tracer technique. In the single-tracer group, an average of two lymph nodes were removed per patient versus an average of three lymph nodes per patient in the dual-tracer group (*p* = 0.001). No loco-regional recurrences have been reported in either group at a mean follow-up of 34 months. The single-tracer technique can be a reliable method for the axillary management in cN + breast cancer patients undergoing neo-adjuvant chemotherapy and achieving nodal disease regression (ycN0), in terms of loco-regional recurrences. This demonstrates that even if fewer sentinel lymph nodes are identified using the single tracer technique (and potentially the false negative rate of the procedure is higher), this does not impact on the rate of loco-regional recurrences. However, longer follow-up is required to confirm these findings.

## Introduction

The management of the axilla in patients with breast cancer is a crucial aspect of oncological treatment, and sentinel lymph node biopsy (SLNB) has become the standard technique for axillary staging in most patients with early-stage breast cancer. In patients with negative lymph nodes, sentinel lymph node biopsy was validated in 1994 by Giuliano et al. [1] as a reliable technique.

In recent years, SLNB has replaced ALND as the staging tool for women undergoing upfront surgery, without compromising overall survival (OS) or disease-free survival (DFS). The therapeutic use of ALND for patients with clinically occult sentinel lymph node metastases has also been debated. Current national and international guidelines suggest that completion ALND can safely be avoided in selected patients with up to two metastatic sentinel lymph nodes during upfront breast-conserving surgery [2].

However, significant disparities still exist in axillary management between and within countries [3, 4], particularly regarding patients undergoing NAC who present with nodal disease and subsequently become cN0 after systemic treatment. For such patients, it has been demonstrated that SLNB alone after NAC results in an FNR > 10% [5–7], while evidence shows that removing three or more SLNs reduces the FNR below 10%.

For patients presenting with positive lymph nodes and undergoing neo-adjuvant chemotherapy, modifications to the technique have been introduced to reduce the (FNR) associated with it.

Both the SENTINA and ACOSOG Z1071 trials demonstrated that the likelihood of identifying three or more SLNs was significantly higher when dual-tracer techniques were used [7, 8]. However, the dual-tracer technique was employed in only 28% of patients in SENTINA and 79% in ACOSOG Z1071, with three or more SLNs removed in just 34% and 57% of cases, respectively. In a recent study conducted at Memorial Sloan Kettering Cancer Center (MSKCC), where all patients underwent SLNB with a dual-tracer technique, three or more SLNs were removed in 93% of cases. In this study, the only two factors associated with a lower likelihood of identifying three or more SLNs were higher BMI and the presence of lymphovascular invasion [9].

Thus, the safety and feasibility of SLNB after NAC remain controversial. Some studies have reported an FNR exceeding the generally accepted cutoff of 10%, and as a result, some guidelines still recommend ALND for these patients [10].

To further reduce the FNR in cN + patients, several strategies have been proposed, including targeted axillary dissection (TAD), which involves removing the marked metastatic lymph node prior to NAC in addition to the sentinel lymph node. The metastatic lymph node is marked using various techniques (carbon tattooing, radioactive iodine, metal clips, ferromagnetic seeds, etc.).

This approach has demonstrated a FNR of 1.4%, suggesting that TAD could improve the success of SLNB while reducing long-term morbidity.

Despite the increasing use of TAD in clinical practice, data from prospective studies remain limited. Some studies have highlighted limitations in the visibility and identification of clipped lymph nodes after NAC, suggesting that clip placement may not be suitable for routine clinical use [11].

Another technique, tailored axillary surgery (TAS), involves SLNB combined with the removal of palpable suspicious lymph nodes, thereby tailoring the extent of axillary surgery to the extent of axillary disease. However, the definition of palpable suspicious lymph nodes is arbitrary, and TAS remains a pragmatic concept left to the surgeon’s discretion. Nonetheless, TAS has shown promise, as it selectively reduces the number of positive lymph nodes while being far less radical than ALND [12].

In Germany, the 2019 S3 guidelines still recommend complete ALND for patients with initial lymph node involvement. However, the German Working Group Gynecological Oncology (AGO) updated its recommendations in 2019, endorsing TAD as the preferred technique for this patient subgroup. In other European countries, such as Sweden, Norway, and Finland, ALND remains the standard of care for these patients. In Italy, most patients undergo SLNB using the dual-tracer technique without marking and removing the target lymph node (TLN) [13, 14].

The aim of our study is to evaluate the efficacy and safety of the single-tracer technique in identifying sentinel lymph nodes and compare this approach with the dual-tracer technique. Specifically, we focus on the number of lymph nodes identified using the two techniques and whether this impacts the long-term loco-regional recurrence rate rather than the false negative rate.

## Materials and methods

We retrospectively collected data at the Malzoni Cancer Center in Avellino from January 2020 to December 2021 and at the Breast Unit of the Federico II University Hospital in Naples from January 2022 to January 2024. We included consecutive patients diagnosed with cN + breast cancer who underwent neoadjuvant chemotherapy and achieved nodal disease regression (ycN0). Patients were staged before and after chemotherapy using breast ultrasound, mammography, magnetic resonance imaging, PET-CT, lesion biopsy, and fine-needle aspiration cytology (FNAC) of lymph nodes.

The patients were divided into two groups: “dual tracer” technique for sentinel lymph node identification and “single tracer” technique, according to the period of time they have been treated. Those enrolled at the Malzoni Cancer Center underwent single tracer technique and those enrolled at the Breast Unit Federico II underwent the dual tracer technique.

In the single-tracer technique, patients were marked within 12 h prior to surgery with a radioactive tracer (technetium-99 microalbumin) injected near the tumor site. The radioactive tracer accumulates in the sentinel lymph nodes, emitting radiation detected intraoperatively using a gamma probe, which converts the radiation into numerical values to identify and precisely remove the lymph nodes.

The dual-tracer technique, on the other hand, involves the use of two tracers: a radioactive tracer (technetium-99 microalbumin) and a dye (indocyanine green, ICG). The ICG was injected directly into the breast in the subcutaneous periareolar or retroareolar tissues. The amount injected was ≥ 2 mL (5 mg/mL). After the injection, a short breast massage followed. The time between injection and incision did not exceed 10 min. Sentinel lymph nodes were located using a fluorescence imaging system (OrbEye, Olympus).

The inclusion criteria for our study required a first diagnosis of breast cancer (confirmed by core biopsy), cN1–cN2 (confirmed by core biopsy/fine-needle aspiration or the presence of highly suspicious lymph node(s) on imaging), cT1–cT2, patients scheduled for neo-adjuvant systemic therapy, male or female patients aged ≥ 18 years, and signed informed consent.

Exclusion criteria included cT3–cT4c, cN3–cN4, distant metastases, recurrent breast cancer, inflammatory or extra-mammary breast cancer, bilateral breast cancer, a history of invasive breast cancer, ductal carcinoma in situ (DCIS), or any other invasive cancer, confirmed or suspected metastases of supraclavicular or internal mammary lymph nodes, axillary surgery prior to NAC (e.g., SLNB or nodal sampling), pregnancy, or patients not eligible for surgical treatment.

Statistical analysis was performed with IBM SPSS Version 26 (IBM Corp., Armonk, N.Y.). Continuous variables are reported as mean ± standard deviation and were compared by the Mann–Whitney *U* test; categorical variables are reported as percentages and compared by the Fisher’s exact test. A value of *p* ≤ 0.05 was considered statistically significant.

A power analysis has been conducted to avoid interference of small sample size on the outcomes; Cohen’s d has been determined by calculating the mean difference between your two groups and then dividing the result by the pooled standard deviation; a Cohen’s D of 0.3, 0.5 and 0.8 corresponds to mild, moderate and large effect sizes, respectively.

The primary endpoint of this study was the identification rate (IR) of sentinel lymph nodes (SLNs) using the single-tracer versus dual-tracer technique in patients with cN + breast cancer down-staged to ycN0 after NAC. Secondary endpoints included the average number of SLNs identified and removed; the number of ALNDs performed based on intraoperative findings; the oncological outcomes in terms of local recurrence, loco-regional recurrence, and axillary recurrence at follow-up.

ALND was performed in cases of macrometastases or micrometastases depending on breast pathological response (pCR), while SLNB alone was accepted in patients with negative SLNs. In patients with residual disease in SLNs but complete pathological response in the breast, the decision regarding ALND or radiotherapy was discussed in a multidisciplinary setting.

## Results

Of the 76 patients in our sample, 42 underwent sentinel lymph node removal using the dual-tracer technique, while 34 underwent the single-tracer technique.

Sentinel lymph node identification was successful in 100% of patients in both the single- and dual-tracer groups.

The average age of the single-tracer group was 42 ± 6 years, while the dual-tracer group had an average age of 55 ± 1 years, including one male patient. Patients’ baseline characteristics are reported in Table 1.

**Table 1:** Patients’ baseline characteristics

	Dual Tracer (*n* = 42)	Single Tracer (*n* = 34)	p
*Age, years* (*mean ± SD*)	42 ± 6	55 ± 1	0.001
*T*, *n* (%)			0.68
1	13 (31%)	12 (35%)	
2	29 (69%)	22 (65%)	
*N*, *n* (%)			0.37
1	30 (71%)	21 (62%)	
2	12 (29%)	13 (38%)	
*Biology*, *n* (%)			0.88
HER2 -	8 (19%)	8 (23.5%)	
HER2 +	15 (37.5%)	11 (32%)	
Triple negative	19 (43.5%)	15 (44.5%)	

Breast-conserving surgery was performed in 55 patients, while 21 underwent bilateral mastectomy due to a positive BRCA1-2 genetic test, with contralateral risk-reduction mastectomy.

Patients were followed up semi-annually for an average of 34 months (range: 10–58 months), with no loco-regional recurrences reported to date.

In the single-tracer group, an average of two lymph nodes were removed per patient (2 ± 1). In contrast, the dual-tracer technique identified and removed an average of three lymph nodes per patient (3 ± 1) (*p* = 0.001). No loco-regional recurrences have been reported in either group to date (Table 2).

**Table 2:** Axillary treatment in both groups

	Dual tracer (*n* = 42)	Single tracer (*n* = 34)	*p*
*SLN removed (mean ± SD)*	3 ± 1	2 ± 1	0.001
*Patients with metastatic SLN, n. (%)*			0.81
Macro-metastatic	5 (12%)	6 (17.6%)	
Micro-metastatic	9 (21.4%)	9 (26.5%)	
ALND, *n* (%)	12 (28.6%)	15 (44%)	0.16
Lymph nodes removed during ALND (mean ± SD)	12 ± 3	12 ± 4	0.9
Positive lymph nodes after ALND (mean ± SD)	4 ± 2	5 ± 3	0.08
RNI	2	0	0.68

*SLN* sentinel lymph node, *ALND* axillary lymph node dissection, *RNI* regional node irradiation

All patients with micro- or macro-metastatic SLNs at frozen sections have been treated with ALND.

In the dual-tracer group, five patients had macro-metastases detected intraoperatively and underwent ALND. Micro-metastases were identified in nine patients, seven of whom underwent ALND (those who did not achieved a complete response at the level of the breast), while two did not undergo ALND (those who achieved a pathological complete response (pCR) in the breast. The remaining 28 patients, whose sentinel lymph nodes were free of neoplasia, did not undergo lymphadenectomy. In the dual-tracer group, 12 ALNDs were performed, with an average removal of 12 lymph nodes, and only four cases revealed additional metastatic lymph nodes (Table 2).

In the single-tracer group, nine patients exhibited micro-metastases during intraoperative examination, while six had macro-metastases. All these patients underwent ALND, resulting in a total of 15 lymphadenectomies. However, additional metastatic lymph nodes were identified in only five cases (all in cases with macro-metastases at the level of the sentinel lymph node). The remaining 19 patients had sentinel lymph nodes free of neoplasia during intraoperative examination and did not undergo lymphadenectomy (Table 2).

The treatment plan for each patient with cN + breast cancer scheduled for NAC was reviewed by the multidisciplinary oncology team, which included breast surgeons, medical oncologists, pathologists, radiation oncologists, radiologists, geneticists, and plastic surgeons. Patients with triple-negative breast cancer received Anthracyclines and Taxanes in combination or not with Carboplatin or Pembrolizumab according to patients’ and cancer characteristics. Adjuvant therapy included Pembrolizumab or capecitabine according to patients’ and cancer characteristics and response to therapy.

Patients with luminal HER2-positive tumors were treated with Anthracyclines, Cyclophosphamide, and Taxanes with Trastuzumab and Pertuzumab in the neoadjuvant setting. Adjuvant therapy continued with HP for patients with pCR, TDM1 for patients with residual disease. Patients with luminal HER2-negative tumors received Anthracyclines and Taxanes as neoadjuvant therapy, followed by hormone therapy in the adjuvant setting. In patients with high Ki-67, CDK inhibitors were also used. Regarding hormone therapy, premenopausal patients were treated with Tamoxifen and LH-RH analogs, while postmenopausal patients received Aromatase Inhibitors. BRCA1/2-mutated patients with residual disease also received Olaparib.

For all patients, the surgical treatment was performed within 3 and 4 weeks after the final administration of chemotherapy. After NAC, before surgery, patients were restaged to assess the response to systemic treatment and the residual tumor size.

Patients who underwent breast-conserving surgery received adjuvant breast radiotherapy. The two patients who had only one micro-metastasis on sentinel lymph node biopsy and showed complete pathological response in the axillary region did not undergo axillary lymph node dissection (ALND), but rather received targeted nodal irradiation (RNI).

A subgroup analysis, based on tumor biology, also has been carried out.

Among patients with HER2-negative breast cancer, only the number of ALND significantly differs between single tracer and double tracer group (6 vs 2, *p* = 0.04); among patients with HER2-positive breast cancer, single tracer groups have a significantly higher number of lymph nodes removed during ALND (13.6 ± 1.15 vs 11.75 ± 1.7, *p* = 0.004). Finally, among patients with TN breast cancer, there was a significantly higher number of SLN harvested on double tracer group (3.2 ± 0.97 vs 1.93 ± 0.8, *p* = 0.001) and a significantly higher number of positive lymph nodes after ALND in single tracer group (4.5 ± 1.64 vs 3.3 ± 1.21, *p* = 0.02). Data analysis has been summarized in Table 3.

**Table 3:** Analysis based on tumor biology

	Single tracer	Double tracer	*p*
HER2 – (n. of patients)	8	8	
SLN removed (mean ± SD)	2.4 ± 0.74	3 ± 0.92	0.17
Macrometastatic, *n*	3	1	0.24
Micrometastatic, *n*	3	1	0.24
ALND, *n*	6	2	0.04
Lymph nodes removed during ALND (mean ± SD)	12 ± 3.57	13 ± 1.41	0.47
Positive lymph nodes after ALND (mean ± SD)	4.83 ± 2.48	3 ± 0.1	0.06
*HER2* + *(n. of patients)*	11	15	
SLN removed (mean ± SD)	2.1 ± 0.53	2.6 ± 0.97	0.13
Macrometastatic, *n*	1	2	0.73
Micrometastatic, *n*	2	2	0.73
ALND, *n*	3	4	0.97
Lymph nodes removed during ALND (mean ± SD)	13.6 ± 1.15	11.75 ± 1.7	0.004
Positive lymph nodes after ALND (mean ± SD)	6.6 ± 2.08	5.5 ± 2.08	0.19
*Triple-Negative (n. of patients)*	15	19	
SLN removed (mean ± SD)	1.93 ± 0.8	3.2 ± 0.97	0.001
Macrometastatic, *n*	2	2	0.8
Micrometastatic, *n*	4	6	0.75
ALND, n	6	6	0.6
Lymph nodes removed during ALND (mean ± SD)	11 ± 5.51	12 ± 2.09	0.47
Positive lymph nodes after ALND (mean ± SD)	4.5 ± 1.64	3.3 ± 1.21	0.02

## Discussion

The indications for NAC in breast cancer treatment have evolved over time [15]. Currently, NAC is administered for locally advanced and inoperable cancer enabling less-invasive surgical procedures, converting up to 75% of cases from mastectomy to breast-conserving surgery (BCS) [16, 17], and decreasing the need for axillary lymph node dissection (ALND) by over 40% [18, 19], but it is also administrated for smaller tumors, according to their biology (triple-negative or HER2-positive cancers) [20, 21].

In our study, we observed that despite axillary surgical axillary de-escalation, there were no loco-regional recurrences at a mean follow-up of 34 months. This demonstrates that even if fewer sentinel lymph nodes are identified using the single tracer technique (and potentially the false negative rate of the procedure is higher), this does not impact on the rate of loco-regional recurrences. Adjuvant therapies, rather than surgery, will determine the clinical course of the disease.

As mentioned before, in the ACOSOG Z1071 study, the IR using dual tracers was 93.8%, and it was only 88.9% when a single radioactive tracer was used, with the use of single tracer being the technique with the lowest SLN IR (78.6%) [7, 8]. The same statement was made by Kuehn et al. [6] in the SENTINA study, showing that the use of dual tracer was associated with a significant increase in the detection rate (87.8% vs. 77.4% with radioisotope alone) in cN + patient turning into ycN0, resulting in a higher number of nodes being detected.

In contrast, a meta-analysis performed by Mok et al. [22] in 2019 showed that the pooled IR for dual-tracer SLN mapping (RI + BD) was 96.7%, a value extremely close to their obtained pooled IR for single tracer, i.e., 96.5%.

Our findings also align with those of Galimberti et al. [23]. The single tracer technique was used by Galimberti et al. in a cohort of patients initially cN + , subsequently down-staged to cN0 after NAC, demonstrating no axillary recurrences at a mean follow-up of 10 years. Furthermore, the study performed by Tinterri et al. [24] regarding the follow-up of patients who became or remained cN0 after NAC and underwent SLNB-alone confirms that the status of SLN remains a significant prognostic factor in these patients. They compared ALND with SLNB in cN + patients down-staged to ycN0 after NACT, reporting axillary recurrence rates for SLNB ranging between 0.6% and 2.1%.

Cavalcante et al. [25], using methylene blue dye in cN + patients who converted to ycN0 after NAC, showed, similarly to our study, no loco-regional recurrences. Five-year disease-free and overall survival were, respectively, 85.9% (95%CI: 74–99.8) and 96.3% (95%CI: 89.4–100).

In contrast, the NEOSENTITURK MF-1803 study compared sentinel lymph node biopsy using methylene blue dye and targeted axillary dissection (TAD) in the same patient population—cN + converting to cN0 after NACT. A total of 976 patients with cT1-4N1-3M0 disease underwent SLNB (*n* = 620) or TAD alone (*n* = 356). Overall, no difference was found between patients treated with TAD and patients treated with SLNB in the median number of total lymph nodes removed (TAD, 4 [3–6] vs SLNB, 4 [3–6]; *P* = 0.09). Among patients with ypN-positive disease, those who underwent TAD were more likely to have a lower median lymph node ratio (TAD, 0.28 [IQR, 0.20–0.40] vs SLNB, 0.33 [IQR, 0.20–0.50]; *P* = 0.03). At a median follow-up of 39 months (IQR, 29–48 months), no significant difference was found in the rates of ipsilateral axillary recurrence (0.3% [1 of 356] vs 0.3% [2 of 620]; P ≥ 0.99) or loco-regional recurrence (0.6% [2 of 356] vs 1.1% [7 of 620]; *P* = 0.50) between the TAD and SLNB groups, with an overall loco-regional recurrence rate of 0.9% (9 of 976). The initial clinical tumor stage, pathologic complete response, and use of blue dye alone as a mapping procedure were not associated with the outcome. Even though patients with TAD demonstrated an increased disease-free survival rate compared with the SLNB group, this difference did not reach statistical significance (94.9% vs. 92.6%; *P* = 0.07). Factors associated with decreased 5-year disease-specific survival were cN2-3 axillary stage (cN1, 98.7% vs. cN2-3, 96.8%; *P* = 0.03) and non-luminal type tumor pathologic characteristics (luminal, 98.9% vs. non-luminal, 96.9%; *P* = 0.007) [26].

Historically, SLNB was not recommended as an alternative to ALND in cN + breast cancer patients down-staged to ycN0 after NAC due to earlier studies reporting high false-negative (FN) rates for SLNB in this cohort, often exceeding 10% [6, 27–29].

These studies, however, focused more on the diagnostic accuracy of procedures rather than prognostic outcomes. In contrast, our study aims to evaluate oncological outcomes in terms of loco-regional recurrences.

In our study, we omitted ALND in patients with micro-metastases at the level of the sentinel lymph node, provided that the patient had a clinical complete response. Even in these cases, no recurrences were observed, which is an important finding despite a very small patient cohort.

As proposed in the NEONOD trial [30], it may become feasible to omit ALND in this subset of patients as already suggested by the ICARO study for isolated tumor cells (ITCs) [31].

These findings support the hypothesis that the extent of axillary surgery can be safely reduced in selected patients after NAC. In this context, the ongoing ALLIANCE A11202 trial is particularly relevant. This phase III randomized study compares axillary lymph node dissection with axillary radiotherapy in patients who have positive sentinel lymph nodes after NAC. The results of this study will help determine whether axillary radiotherapy alone can provide equivalent oncological outcomes to ALND while reducing treatment-related morbidity. This study reflects a growing interest in personalizing axillary management and may further support de-escalation strategies in the post-neo-adjuvant setting [32].

In our clinical practice, patients who achieve nodal pathological complete response after NAC (ycN0) are generally not offered regional nodal irradiation, in accordance with the preliminary results of the NSABP B-51 trial, which support the omission of post-mastectomy radiation therapy and regional nodal irradiation (RNI) in this setting. On the other hand, in cases of residual nodal disease, we adopt an intensified systemic treatment approach, including the use of pembrolizumab when indicated, based on current guidelines and evidence from recent trials supporting its role in high-risk triple-negative breast cancer with residual disease after NAC.

The present study has several limitations. First, the retrospective nature of the analysis introduces potential selection bias, particularly regarding the assignment of patients to the single- or dual-tracer group based on the institution where they were treated. Second, the relatively small sample size, especially in the subgroup analyses by tumor biology, may reduce the power to detect statistically significant differences. Third, the median follow-up of 34 months, although promising, may still be insufficient to capture late recurrences. Fourth, the lack of uniform criteria for ALND in micro-metastatic SLNs and the potential variability in imaging interpretation may have introduced heterogeneity in treatment decisions.

Moreover, according to the power analysis, the effect size of the study is small and the power is also low.

Finally, although oncologic outcomes appear favorable, further prospective studies with standardized protocols are needed to validate our findings.

Therefore, prospective trials with larger patient samples and longer follow-up periods are necessary.

## Conclusions

In our clinical experience, we have observed that the single-tracer technique can be a reliable method for the axillary management in cN + breast cancer patients undergoing neo-adjuvant chemotherapy and achieving nodal disease regression (ycN0), in terms of loco-regional recurrences. However, further follow-up is required to confirm these findings.

## Data Availability

The data that support the findings of this study are available from corresponding author upon reasonable request due to ethical restrictions.
